# A Coarse-to-Fine Framework for Oil–Water Interface Measurement in Small-Caliber Transparent Test Tubes

**DOI:** 10.3390/s26113555

**Published:** 2026-06-03

**Authors:** Bo Zhou, Yang Zhou, Jigang Zou, Zhandong Lv, Weijie Zhang, Ruihan Wang, Shengwei Meng

**Affiliations:** 1 State Key Laboratory of Continental Shale Oil, Exploration, Development Research Institute of Daqing Oilfield, Daqing 163712, China; 2 Harbin Institute of Technology, School of Electronics and Information Engineering, No.92 Xidazhi Street, Harbin 150001, China

**Keywords:** liquid-surface measurement, oil–water interface, coarse-to-fine framework, machine vision

## Abstract

Accurate oil–water interface measurement in small transparent test tubes is important for subsequent volume readout in laboratory analysis. However, manual observation and conventional vision-based methods are easily affected by illumination variation, wall stains, and bubbles, while deep learning detectors alone usually provide only coarse semantic perception. To address this issue, a coarse-to-fine framework is proposed for robust oil–water interface measurement. In the coarse stage, YOLOv8n is used to provide semantic constraints for subsequent processing. In the fine stage, a Fisher-discriminative chromatic-weighted brightness feature is constructed from RGB information, where the RGB weights are derived from the Fisher criterion to enhance oil–water chromatic separability rather than using fixed grayscale or empirical channel weights. This feature is then fused with a SobelY-based vertical-gradient feature to improve interface localization. A stain-aware row-aggregation strategy with effective-pixel compensation is further introduced to suppress artefact interference. The validated interface position is finally converted into a volume readout, with additional correction for bubble-induced bias. The framework was validated on sampled frames from a complete shale-oil core pressing process conducted under mixed-lighting conditions. Stage-wise evaluation and ablation results indicate that the proposed design improves readout stability under stains, bubbles, and illumination variation, achieving a mean absolute error of 0.0159 mL and keeping the maximum error below 0.03 mL in the current experimental setup.

## 1. Introduction

In petrochemical analysis, sample pretreatment, and other small-volume liquid-handling tasks, accurate identification of the oil–water interface in transparent test tubes is essential for subsequent volume readout and quantitative analysis. In routine laboratory practice, however, such readout is still often performed manually and is therefore susceptible to operator experience, viewing angle, visual fatigue, and ambient illumination [1,2,3]. In typical shale-oil core pressing experiments, the evolving oil–water interface must be repeatedly monitored over time to determine the volume of expelled fluids, requiring operators to manually read the interface position against tube graduation marks at multiple time points. Under high-throughput petrochemical workflows, such repeated visual inspection introduces several operator-dependent limitations. Visual fatigue accumulated during prolonged observation of narrow small-caliber tubes under varying illumination conditions increases the risk of interface misidentification and scale-reading errors, while the cylindrical tube wall and liquid meniscus further introduce parallax error by distorting the apparent interface position unless observations are conducted from an exact and repeatable viewing angle. In addition, measurements performed by different technicians may exhibit inter-operator variability, reducing data repeatability and cross-experiment comparability. The difficulty is further amplified in small-caliber transparent tubes, where the observable interface region is narrow and the graduation interval is compact [4,5], while the curved tube wall further distorts visual appearance [6,7]. Therefore, an automated non-contact vision-based measurement method is required to reduce operator-dependent bias and provide consistent and traceable measurements throughout the experimental process.

To improve efficiency and repeatability, researchers have explored a variety of liquid-surface and interface measurement methods. Non-visual sensing approaches, such as capacitive and microwave-based techniques, can provide liquid-state information without direct contact [8,9,10], but they usually require dedicated hardware and are less convenient for compact transparent laboratory vessels. Consequently, image-based non-contact measurement has become an attractive alternative.

Early vision-based methods mainly relied on grayscale transitions, edge extraction, threshold segmentation, or geometric features to locate liquid levels or calibration marks in transparent containers [6,11]. Although simple to deploy, these methods are easily disturbed by reflections and refractions. Their performance further degrades in the presence of stains, bubbles, and non-uniform illumination [5,7], particularly when the target boundary is narrow and visually weak. Recent studies have moved from hand-crafted image processing toward data-driven visual perception. Existing work has covered automated liquid-handling inspection [1,3], low-light transparent-container detection [2], non-contact visual measurement in transparent vessels [4,5], and liquid–liquid separation workflows [6,7]. These studies show that computer vision is already useful for laboratory perception, but they do not yet resolve metrological readout of a narrow liquid–liquid interface in a small transparent tube.

These studies are related, but they leave the present task unresolved. First, many existing methods focus on air–liquid boundaries [1,12,13] or broader container monitoring and workflow inspection [2,3,14], rather than precise localization of a narrow liquid–liquid interface in a small-caliber tube. Unlike an air–liquid boundary mainly governed by meniscus/air contrast, the oil–water interface in a narrow transparent tube is also affected by phase color difference, cylindrical refraction, bubble overlap, and stain-induced false edges. Second, the combination of a transparent vessel and a small caliber make the problem substantially harder, because curved-wall distortion and reflections interfere with interface visibility [4,6], while stains and bubbles further degrade visual reliability [5,7]. Third, the goal here is not merely to detect an interface-related region, but to support final quantitative readout; the localized interface must therefore be converted into a reliable volume value consistent with laboratory reading practice [4,11,15].

The difference matters here because the target is not coarse phase recognition, but interface-based quantitative readout. Most reported systems are designed for air–liquid estimation [5,12,13], process monitoring or quality inspection [3,14,15], or robotic handling within laboratory workflows [1,2]. Even in liquid–liquid scenarios, the emphasis is usually placed on separation status, phase recognition, or extraction procedures [2], rather than on metrological interface readout for final measurement. Moreover, deep learning detectors are effective for coarse semantic perception [1,5,12], but bounding boxes or region-level predictions alone do not provide the precision required for interface-based readout [7].

To address these issues, this study proposes a coarse-to-fine framework for oil–water interface measurement in transparent small-caliber test tubes (tubes, Tianjin Tianke Glassware Manufacturing Co., Ltd., Tianjin, China). In this framework, the coarse stage provides the tube ROI and disturbance-related cues, while the fine stage performs readout-oriented interface localization within the constrained ROI.

1. A task-oriented coarse-to-fine measurement framework is developed for narrow oil–water interface readout in small-caliber transparent tubes.
2. A Fisher-discriminative chromatic weighting strategy is introduced to strengthen oil–water photometric separability under the adopted imaging setup.
3. A stain-aware fusion-and-validation mechanism is designed to improve interface localization stability and downstream volume readout consistency.

The remainder of this paper is organized as follows. Section 2 presents the proposed method. Section 3 describes the experimental setup, dataset construction, and evaluation protocol. Section 4 reports the experimental results, stage-wise analysis, and ablation study.

## 2. Materials and Methods

### 2.1. System Overview

The proposed system follows a hierarchical coarse-to-fine framework to robustly localize the oil–water interface and report a volume readout from small-caliber glass test tubes (tubes, Tianjin Tianke Glassware Manufacturing Co., Ltd., Tianjin, China). The design is motivated by recent work showing that deep models are effective for coarse liquid-state perception in laboratory vessels, whereas fine boundary readout still benefits from signal-level analysis and geometry-aware post-processing [16,17,18,19]. Accordingly, the system is organized such that semantic perception provides the measurement context, and pixel-level feature analysis determines the final interface row.

In the coarse stage, a lightweight YOLOv8n detector performs multi-class inference on each frame, producing four outputs: the tube ROI, liquid-surface cues, oil bubbles, and stain artefacts. The tube bounding box is used to crop the ROI and suppress irrelevant background structures. The detected liquid-surface cues serve as a semantic constraint for the final interface row, while bubble and stain detections explicitly mark common sources of false edges that would otherwise destabilize classical edge-based approaches. This design is consistent with recent studies in liquid-surface perception and small-object visual inspection, where YOLO-based detectors are mainly used to provide robust semantic priors rather than direct metrological readouts [20,21,22,23].

In the fine stage, the cropped ROI is processed to estimate the interface row in pixel coordinates. After smoothing and grayscale conversion, two complementary cues are constructed: a chromatic-weighted brightness feature to emphasize photometric contrast between oil and water, and a vertical-gradient feature obtained by ROI-level mean centering followed by SobelY-based gradient magnitude extraction. Both cues are standardized at the ROI level and fused pixel-wise into a single response score. Stain detections are converted into a binary mask; pixels inside stain regions are treated as unreliable and excluded from aggregation. The fused feature is then accumulated row-wise to form a one-dimensional response profile, with an effective-pixel compensation applied so that rows with fewer valid pixels are not unfairly suppressed. Candidate interface rows are ranked by the compensated response, and the final interface row is selected as the best-ranked candidate consistent with the YOLO-detected liquid-surface cue region; candidates outside the cue region are rejected in favor of the next-ranked ones. The overall framework follows the logic of combining robust visual detection with edge or gradient-sensitive refinement that has been reported in recent liquid-surface, meniscus, and sub-pixel measurement studies [24,25,26].

**Figure 1:**
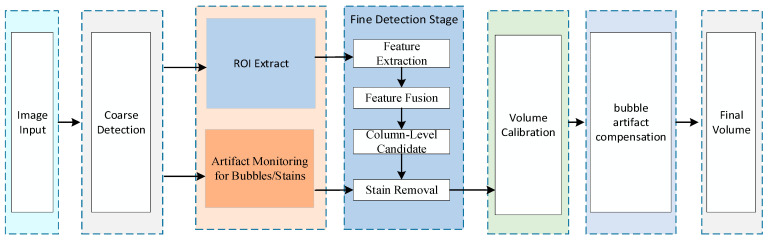
Block Diagram of the System Overview

Finally, the detected interface row is converted into a raw volume reading using graduation anchors. Once the setup is fixed, the graduation marks are manually annotated, and the reading is obtained by piecewise interpolation between adjacent marks. Bubble-induced bias is then compensated by using the coarse-stage bubble detections to calculate the total bubble bounding-box area and applying a proportional correction. Similar strategies for graduation-guided readout, refraction-aware correction, and bubble-related compensation have been reported in recent machine-vision measurement studies and oil-filled-equipment monitoring tasks [27,28,29,30]. Figure 1 summarizes the overall workflow of the proposed measurement system.

### 2.2. Coarse Semantic Perception Stage

#### 2.2.1. YOLOv8n Architecture

The coarse stage relies on YOLOv8n, selected not as a default deep learning choice, but as a pragmatic response to the geometric and optical constraints imposed by small-caliber glass test tubes. In this measurement scenario, the tube structure occupies a narrow image region, visual cues are weak and fragmented, and the imaging environment is subject to reflection, partial occlusion, and illumination drift. Any detector used at this stage must satisfy three simultaneous requirements: reliable localization under optical disturbance, sensitivity to small and elongated structures, and sufficiently low computational overhead to support real-time processing. Recent reviews and application studies have shown that the YOLO family offers an effective balance between efficiency and accuracy, especially in scenes dominated by small or visually degraded targets [20,21,22,23].

Traditional edge-based approaches—such as Canny edge detection and Hough-based line extraction—have historically been applied to tube-like structures and remain conceptually appealing due to their simplicity [24,25]. However, their effectiveness depends strongly on the presence of continuous, high-contrast boundaries. In the present context, tube edges are frequently interrupted by reflections, stains, internal fluid patterns, or variations in glass thickness. These conditions compromise the assumptions underpinning classical edge extraction, motivating the adoption of a learning-based detector that can absorb such variability during training. Similar limitations of purely handcrafted edge systems have also been noted in low-contrast and glass-vessel measurement studies [26,29].

YOLOv8n fulfills these requirements through its compact CSP-based backbone and decoupled detection head, which together enable efficient multi-scale feature extraction while maintaining a lightweight model footprint [20,21]. Unlike heavier variants in the YOLOv8 family, the “n” configuration preserves detection stability without imposing unnecessary computational cost, making it suitable for continuous laboratory operation where latency and hardware constraints matter. This architectural choice is consistent with the role of the coarse stage, where fast and stable localization is more important than pixel-level metrological precision. More importantly, the model learns semantic and structural features directly from annotated tube images, allowing it to infer tube boundaries even when edges are incomplete, blurred, or visually distorted by the cylindrical glass wall. Thus, the detected tube region provides a structural prior that constrains the subsequent measurement process to a physically meaningful area, reducing the dependence on fragile hand-crafted edge or geometry assumptions. This preference for a lightweight detector is also supported by recent small-object and liquid-recognition studies based on improved YOLO architectures [22,23].

From an algorithmic standpoint, the role of YOLOv8n in the proposed system is deliberately limited. Its compact backbone reduces computational burden, while its detection head provides bounding-box-level spatial constraints for tube regions and disturbance-related cues. The detector is not expected to provide metrological precision, nor to resolve the oil–water interface at pixel level. Its task is instead to supply stable clues that confine subsequent fine-stage analysis to the physically meaningful measurement area. By operating at the semantic level rather than relying on explicit geometric assumptions, YOLOv8n offers a robust entry point for downstream processing without entangling coarse localization with fine measurement, which is consistent with the division of labor adopted in recent liquid-surface and meniscus-detection systems [16,17,18].

**Figure 2:**
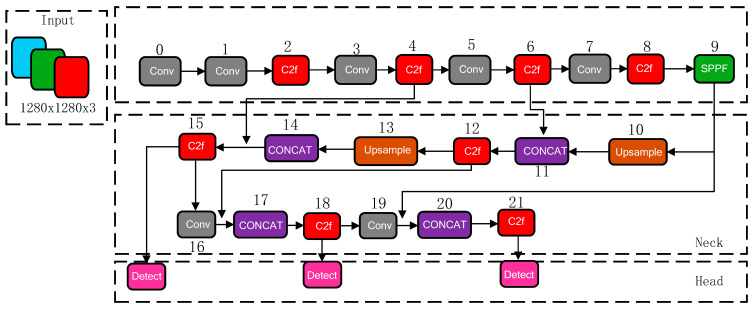
Network architecture of the YOLOv8n-based coarse detector.

For these reasons, YOLOv8n is employed as the coarse detection backbone in the proposed framework. Its outputs serve as geometric and semantic priors, while precision is deferred to the fine-stage processing described in subsequent sections, see Figure 2.

#### 2.2.2. Detection Targets and Label Definition

**Figure 3:**
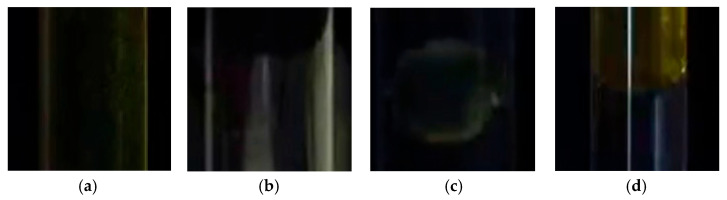
Typical appearance examples of the four annotated categories in the collected tube images: (**a**) tube ROI; (**b**) stain artefacts on the tube wall; (**c**) oil bubbles near the interface; (**d**) liquid-surface cues around the oil–water boundary.

The coarse detector is trained to predict four categories because each of them directly supports a downstream decision in the measurement system. The Tube ROI bounds the valid measurement region and provides a consistent coordinate frame for subsequent processing. Liquid-surface cues correspond to weak horizontal patterns that repeatedly appear around the true interface; they are used as a semantic constraint so that the final interface row must fall within the cue-consistent band. Stain artefacts represent tube-wall contamination that often produces strong but irrelevant edge responses. Similar task-oriented label decomposition has been shown to improve robustness in recent YOLO-based liquid-surface, infusion-bottle, and meniscus-recognition studies, see Figure 3.

These outputs provide auxiliary priors for the fine stage: the stain mask marks invalid regions to be ignored, while the detected interface bubbles are summarized by their overall box area to support a subsequent compensation in the final volume readout. Recent work on bubble detection and compensation further suggests that explicitly modeling bubble-induced disturbance is preferable to treating bubbles only as generic noise [27,28], see Table 1.

#### 2.2.3. Training Strategy and Data Augmentation

The YOLOv8n detector was implemented using Ultralytics YOLO v8.3.235 and trained on images of small-caliber test tubes collected under typical laboratory conditions. All images were manually annotated with four categories—tube ROI, liquid-surface cues, oil bubbles, and stain artefacts—following consistent bounding-box labeling rules. The training set includes variations in liquid color, tube type, camera angle, internal reflections, and illumination intensity to ensure adequate coverage of real measurement scenarios. This data organization follows the common practice in recent vision-based liquid-surface and laboratory-inspection studies, where annotation diversity is essential for robustness under illumination and container variability [17].

The dataset was further organized to include representative illumination variations commonly encountered in practical laboratory environments, including bright, low-light, and spatially non-uniform lighting conditions.

Under bright illumination conditions, the tube is illuminated by the primary laboratory ceiling lights, resulting in an illuminance of approximately 500–800 lux at the tube surface without additional shadowing. Images captured under this condition exhibit high overall brightness and a relatively uniform background.

Under low-light illumination conditions, the primary ceiling lights are turned off or dimmed, and illumination is provided only by ambient light from adjacent workstations or monitor screens, corresponding to approximately 50–150 lux. Images captured under this condition exhibit low global brightness and may contain localized dark regions.

Under mixed illumination conditions, the tube is partially shadowed by surrounding equipment or the camera fixture, producing both bright and dark regions within the same image. Illumination is spatially non-uniform, with certain areas of the tube well illuminated while others remain in shadow. The illuminance across the image varies from approximately 100 to 600 lux.

To enhance robustness and reduce overfitting, standard data-augmentation operations were adopted during training. These include random horizontal shifts, mild rotations, brightness and contrast jittering, Gaussian noise injection, and small-scale perspective distortions. Augmentation was intentionally kept moderate to preserve the geometric characteristics of tubular structures while improving the detector’s tolerance to illumination changes and surface irregularities. Similar augmentation policies have been widely used in recent transparent-container and liquid-perception benchmarks to improve generalization without destroying task-relevant geometric cues [13,16,17].

The model was trained using Ultralytics YOLO v8.3.235 and PyTorch v2.8.0, including objectness, classification, and bounding-box regression terms [20,21]. An AdamW optimizer implemented in PyTorch v2.8.0 together with a cosine learning-rate schedule was adopted to ensure stassble optimization. The batch size and input resolution were chosen to balance computational efficiency and detection accuracy. A separate validation subset was used throughout training to monitor performance and mitigate overfitting. The trained detector achieved reliable localization of tube regions and relevant artefacts, providing robust coarse-stage support for the subsequent fine-stage interface detection.

### 2.3. Fine-Stage Interface Localization

#### 2.3.1. ROI Preprocessing

To obtain a clean and stable signal around the oil–water interface, the ROI undergoes a simple photometric preprocessing step. Two operations are applied in a fixed order: a spatial Gaussian blur (The proposed algorithm was implemented using custom-written scripts in Python v3.10.18) with a kernel, followed by conversion from RGB to a single-channel grayscale (The proposed algorithm was implemented using custom-written scripts in Python v3.10.18) image. The goal is to suppress high-frequency noise and small defects on the tube wall while preserving the physically meaningful intensity transition at the interface. Such preprocessing remains common in robust liquid-surface, edge-based interface, and sub-pixel measurement systems because it stabilizes downstream gradient estimation under noisy or low-contrast imaging conditions [17,24,26].

First, a normalized 5×5 Gaussian kernel G(k,l) is used to smooth each color channel of the ROI independently:

(1) $$\tilde{C}(x,y)=\sum_{2}^{k=-2}\sum_{2}^{l=-2}G(k,l)C(x-k,y-l),\;\;C\in \{R,G,B\}$$

The 5×5 kernel is chosen as a practical compromise. It is sufficiently wide to suppress sensor noise and fine scratches on the tube wall, yet still narrow compared with the apparent thickness of the oil–water interface. A smaller kernel would preserve more high-frequency noise and increase false edge responses, whereas a larger kernel would risk blurring the interface transition and reducing localization precision.

The resulting field provides a compact feature in which structural transitions are emphasized, and channel-specific color fluctuations are largely suppressed. This combination of 5×5 Gaussian smoothing and grayscale conversion yields a noise-robust and structurally consistent ROI suitable for subsequent fine-stage analysis of the oil–water interface.

#### 2.3.2. Chromatic-Weighted Brightness Construction

To enhance the photometric contrast at the oil–water interface, a chromatic-weighted brightness feature is constructed from the pre-smoothed RGB ROI. Let the ROI be defined on a discrete grid. After Gaussian smoothing, each pixel is represented in the RGB color space as:

(2) $$I_{\mathrm{RGB}}(x,y)=\left|\begin{array}{c} R(x,y)\\ G(x,y)\\ B(x,y) \end{array}\right|,\;\;(x,y)\in \Omega$$

where R(x, y),G(x, y), and B(x, y) denote the red, green, and blue channel intensities, respectively.

Instead of directly using the standard luminance or a single-color channel, we define a chromatic-weighted brightness feature C(x, y) as a linear combination of the three-color channels. This projection allows us to emphasize those spectral components that exhibit the largest systematic difference between oil and water, while suppressing less informative or more noise-sensitive channels, so that the resulting scalar feature provides a stronger and more stable contrast at the oil–water interface than any individual channel or conventional luminance definition.

(3) $$C(x,y)=w_{R}R(x,y)+w_{G}G(x,y)+w_{B}B(x,y)=w^{T}I_{RGB}(x,y)$$

where $w=[w_{R},\;w_{G},\;w_{B}{]}^{T}$ is a non-negative weight vector. The critical point is that w is determined from the chromatic characteristics of the oil–water interface via an optimization procedure, rather than being chosen heuristically.

To model the color difference across the interface, a small calibration set is constructed from representative images. Around manually annotated interface locations, two groups of pixels are sampled: pixels belonging to the upper oil phase and pixels belonging to the lower water phase. Their RGB vectors are collected into two sets. Using these samples, the chromatic weighting vector is estimated by Fisher linear discriminant analysis, which seeks a projection that maximizes the between-class contrast of oil and water while accounting for within-class covariance.

(4) $$X_{o}=p_{i=1}^{N_{o}},X_{w}=p_{j=1}^{N_{w}}$$

where $p_{i}^{o},\;p_{j}^{w}\in R^{3}$ denote RGB samples from the oil and water regions, $N_{o}$ and $N_{w}$ are the corresponding sample counts.

The mean color vectors of the two phases in RGB space are defined as:

(5) $$\mu^{o}=\frac{1}{N_{o}}\sum_{i=1}^{N_{o}}p_{i}^{o},\;\;\mu^{o}=[\mu_{R}^{O},\mu_{G}^{O},\mu_{B}^{O}]$$

(6) $$\mu^{w}=\frac{1}{N_{w}}\sum_{j=1}^{N_{w}}p_{j}^{w},\;\;\mu^{w}=[\mu_{R}^{w},\mu_{G}^{w},\mu_{B}^{w}]$$

and the pooled within-class covariance matrix is given by

(7) $$S=\frac{1}{N_{o}+N_{w}}\left(\sum_{i=1}^{N_{o}}(x_{i}^{o}-\mu_{o}){\left(x_{i}^{o}-\mu_{o}\right)}^{T}+\sum_{j=1}^{N_{w}}(x_{j}^{w}-\mu_{w}){\left(x_{j}^{w}-\mu_{w}\right)}^{T}\right)$$

When projecting an RGB sample p onto the scalar feature $c=w^{T}x$, the mean features of oil and water become $\mu_{o}(w)=w^{T}\mu_{o}$ and $\mu_{w}(w)=w^{T}\mu_{w}$, and the within-class variance along this direction is $\sigma^{2}(w)=w^{T}Sw$. To maximize the contrast between oil and water while accounting for their within-class variability, we adopt a Fisher-type criterion and seek the chromatic weights.

(8) $$w^{*}=\arg\underset{w}{\max}J(w)=\arg\underset{w}{\max}\frac{{\left(\mu_{o}(w)-\mu_{w}(w)\right)}^{2}}{\sigma^{2}(w)}$$

Since the Fisher ratio is scale-invariant, the maximizer is given by

(9) $$\tilde{w}\propto S^{-1}(\mu_{o}-\mu_{w})$$

which is then projected onto the feasible set. The resulting vector $w^{⋆}$ is obtained offline from the calibration dataset and then kept fixed for all subsequent experiments in this work. The $\mu_{o}$ and $\mu_{w}$ are the mean RGB vectors of oil and water samples, and $S^{-1}$ is the within-class scatter matrix. This direction enhances inter-phase separability while reducing the influence of intra-phase colour variation. After normalization and non-negative projection, the resulting vector was used as the RGB weighting vector, so that colour channels contributing more to oil–water discrimination receive larger weights.

With the optimized chromatic weights $w^{*}$, the final chromatic-weighted brightness feature is computed as

(10) $$C(x,y)=w^{*T}I_{\mathrm{RGB}}(x,y),\;\;(x,y)\in \Omega$$

which encodes both luminance and color cues in a single-channel feature tailored to the oil–water system under the adopted illumination and camera response.

The chromatic weights were calibrated once under standard illumination and then kept fixed in all subsequent experiments. This setting can tolerate moderate illumination variations because the Fisher criterion emphasizes relative chromatic differences between the oil and water phases rather than absolute intensity values, provided that the camera white balance and imaging geometry remain unchanged. However, under extreme illumination changes or when the liquid colours differ substantially from the calibrated system, recalibration of the chromatic weights would be required.

#### 2.3.3. Vertical-Gradient Feature Construction

Within the tube ROI, the oil–water interface typically appears as a narrow horizontal transition band. To capture this structural change, we construct a vertical-gradient feature based on the Sobel operator (The proposed algorithm was implemented using custom-written scripts in Python v3.10.18). This choice is motivated by the continued effectiveness of gradient-based operators in liquid boundary localization, especially when combined with subsequent validation or sub-pixel refinement [24,25,26].

Let I(x, y) denote the grayscale image in the ROI, where x and y represent the column and row indices, respectively. To mitigate global illumination bias inside the ROI, we first perform ROI-level mean centering. The mean intensity is computed as

(11) $$\mu_{I}=\frac{1}{WH}\sum_{x-1}^{W}\sum_{y-1}^{H}I(x,y)$$

and the mean-centered image is obtained by

(12) $$I_{0}(x,y)=I(x,y)-\mu_{I}$$

A vertical Sobel operator is then applied to $I_{0}(x,\;y)$ to extract vertical intensity variations. Using the kernel:

(13) $$K_{y}=\left[\begin{array}{ccc} -1 & -2 & -1\\ 0 & 0 & 0\\ 1 & 2 & 1 \end{array}\right]$$

the vertical-gradient feature is computed as:

(14) $$G_{y}(x,y)=(I_{0}*K_{y})(x,y)$$

Since the interface may produce either positive or negative responses depending on local contrast, we use the gradient magnitude

(15) A(x,y)=G(x,y)y

as the vertical-gradient strength feature.

#### 2.3.4. Feature Fusion and Stain-Aware Row Aggregation

The chromatic cue and the vertical-gradient cue capture complementary aspects of the oil–water interface. The chromatic-weighted brightness emphasizes photometric discontinuities, while the SobelY-derived feature highlights structural transitions. To exploit their agreement while suppressing artefact-driven responses, the two cues are fused at the pixel level and then aggregated along image rows. Stain regions are treated as unreliable measurements and are explicitly excluded from the aggregation, with a compensation term applied to account for the reduced number of valid pixels. Similar cue-combination and reliability-weighting ideas have been used in robust liquid-surface, meniscus, and sub-pixel edge estimation studies [17,18,25,26].

Let C(x, y) denote the chromatic-weighted brightness feature and let A(x, y) denote the vertical-gradient magnitude feature. Because the two features are not naturally on the same numerical scale, we first perform ROI-level standardization for each feature:

(16) $$C(x,y)=\frac{C(x,y)-\mu_{C}}{\sigma_{C}+\varepsilon },A(x,y)=\frac{A(x,y)-\mu_{A}}{\sigma_{A}+\varepsilon }$$

where $\mu_{C},\;\sigma_{C}$ and $\mu_{A},\;\sigma_{A}$ are the mean and standard deviation computed over all pixels in the ROI for C and A, respectively, and ε is a small constant to avoid numerical instability.

A fused feature field is then constructed using a convex combination:

(17) R(x,y)=αC(x,y)+(1−α)A(x,y),α∈[0,1]

where α controls the relative contributions of chromatic and structural features. The fused field yields high response scores at locations where both cues consistently indicate the presence of an interface transition. The fusion weight α was set to 0.5 in the final experiments to balance the chromatic-discriminative cue and the gradient cue. This setting was selected based on validation results under different illumination and disturbance conditions and was kept fixed during testing. In general, increasing α gives more emphasis to chromatic information, which is beneficial when the oil–water colour contrast is clear. Conversely, decreasing α gives more emphasis to the gradient cue, which can improve boundary responses under low-light or reflection-disturbed conditions.

To suppress false responses induced by tube-wall contamination, a stain mask M(x,y) is introduced. Pixels located inside detected stain regions are regarded as unreliable and excluded from the subsequent row-wise aggregation:

(18) $$M(x,y)=\left\{\begin{array}{c} 0,(x,y)\in \Omega_{stain}.\\ 1,oterwise \end{array}\right.,R^{\prime }(x,y)=M(x,y)R(x,y)$$

where $\Omega_{stain}$ denotes the set of stain pixels. The fused feature is then projected into a one-dimensional row profile by row-wise accumulation:

(19) $$S(y)=\sum_{x=1}^{W}R^{\prime }(x,y)=\sum_{x=1}^{W}M(x,y)R(x,y)$$

where W represents the ROI width. However, stain masking reduces the number of valid pixels in contaminated rows. Consequently, the raw row-wise response may decrease simply because fewer pixels contribute to the accumulation, rather than because the interface feature is actually weak or absent. To avoid this unfair attenuation, the number of valid pixels in each row is first computed as

(20) $$N(y)=\sum_{x=1}^{W}M(x,y)$$

The effective-pixel-compensated row response is then obtained by averaging the fused feature over the valid pixels and rescaling it to the full row width:

(21) $$\tilde{S}(y)=\frac{S(y)}{N(y)+\varepsilon }$$

This compensation allows partially contaminated rows to maintain response scores comparable to those of clean rows, provided that sufficient valid pixels remain. Therefore, the proposed strategy suppresses false attenuation caused by stain exclusion while preserving the relative prominence of the true interface response.

After constructing the stain-masked fused feature, we obtain the compensated row profile $\tilde{S}(y)$. The oil–water interface row is first estimated as the row index that maximizes this profile, the most strongly supported horizontal transition under the combined chromatic and gradient evidence. This stain-aware fusion-and-aggregation scheme preserves interface-consistent features while suppressing contributions from stain-contaminated pixels, and the effective-pixel compensation maintains comparability across rows with different numbers of valid pixels.

In spite of this robustness, $\tilde{S}(y)$ may still exhibit occasional spurious maxima caused by residual reflections or other local artefacts. To enforce semantic consistency, we further validate the selected interface row using the liquid-surface cue detections provided by the coarse stage. Let $\Omega_{cue}$ denote the set of pixel locations covered by the YOLO-detected liquid-surface cue bounding boxes after featuring them into the ROI coordinate system. Projecting $\Omega_{cue}$ onto the vertical axis yields an admissible set of rows:

(22) $$y_{\mathrm{cue}}=\{y|\exists x\;\mathrm{such}\;\mathrm{that}\;(x,y)\in \Omega_{\mathrm{cue}}\}$$

To tolerate minor localization jitter, we expand this set by a margin δ:

(23) $$y_{\mathrm{cue}}^{\delta }=\{y|\min_{y_{c}\in Y_{\mathrm{cue}}}|y-y_{c}|\leq \delta \}$$

We then rank the candidate interface rows in descending order of the response score. The final interface row is chosen as the highest-ranked candidate that lies within the admissible cue region; equivalently, it can be expressed as

(24) $$y^{\lambda }=\arg\underset{y\in y_{\mathrm{cue}}^{\delta }}{\max}\tilde{S}(y)$$

In implementation, we scan $y_{\left(k\right)}$ from the top of the ranked list and accept the first $y(k)\in y_{\mathrm{cue}}^{\delta }$; any candidate outside $y_{\mathrm{cue}}^{\delta }$ is discarded and the next-ranked one is evaluated. This post-selection validation step anchors the fine-stage estimate to the coarse semantic cues and reduces the risk that non-interface artefacts dominate the final localization.

Importantly, the fine-stage interface estimation is not treated as an isolated pixel-level result. Instead, it serves as an intermediate feature that will be further integrated with measurement-level constraints and coarse-stage semantic information, forming a complete coarse-to-fine fusion from visual perception to physically consistent output.

### 2.4. Volume Readout

#### 2.4.1. Graduation Mark Annotation

To incorporate structural constraints into the estimation, the geometric relationship between pixel coordinates and physical scale is established using graduation anchors. After the imaging geometry is fixed, the pixel positions of the graduation marks are manually annotated on a representative ROI image. Each graduation line provides a reliable anchor between the printed scale and the image coordinate system. The annotation produces an ordered set of anchor pairs, where the first element is the pixel row coordinate of the kth graduation line in the ROI coordinate system and the second element is the corresponding scale or volume value printed on the tube. The anchors are arranged to follow the monotonic progression along the tube axis, ensuring that adjacent marks define a valid interpolation interval. This anchor-based strategy is consistent with recent graduation-guided liquid reading methods for flasks, vials, and pipetting systems [4,6,19].

#### 2.4.2. Scale Readout via Piecewise Interpolation

Given the interface row estimated from the fused feature in the fine stage, the corresponding volume is obtained through a locally constrained feature defined by adjacent graduation anchors. Let the two adjacent anchors be denoted by the lower and upper marks. The detected interface position is then converted into a continuous scale value using piecewise linear interpolation. This local interpolation strategy avoids imposing a global geometric model over the full tube length; instead, it relies on physically printed graduations as ground-truth references. Similar locally anchored readout strategies have been adopted in recent vision-based volume and liquid-surface measurement studies [4,6,11,19].

(25) $$\hat{v}(y^{\lambda })=v_{k}+\frac{y^{\lambda }-y_{k}}{y_{k+1}-y_{k}}(v_{k+1}-v_{k})$$

In practice, the spacing between adjacent marks is small and the feature is locally smooth, making the piecewise linear form sufficient for stable readout. This process ensures that the interface estimate is not only optimal in the response domain but also consistent with the physical distribution of the measurement system.

#### 2.4.3. Bubble-Induced Bias Compensation

The interpolated readout represents the apparent volume implied by the detected interface position. In bubble-rich scenes, oil-phase bubbles introduce a systematic bias by perturbing the observed liquid region and distorting the interface appearance. In addition to structural constraints, coarse-stage semantic information is further integrated at the measurement level. In particular, bubble detections obtained from the YOLO-based coarse stage are incorporated as disturbance descriptors to refine the final measurement. This design is motivated by recent studies showing that bubble detection and compensation can improve the stability of visual measurements in oil-filled or multiphase systems [27,28].

Let ${\left\{B_{i}\right\}}_{i=1}^{N_{b}}$ be the set of bubble bounding boxes detected within the tube ROI for a given frame. Each box $B_{i}$ is parameterized by its width $w_{i}$ and height $h_{i}$ in pixel units. The overall bubble extent is quantified by the total bounding-box area:

(26) $$A_{b}=\sum_{i=1}^{N_{b}}\left(w_{i}h_{i}\right)$$

This design propagates coarse-level semantic cues into the final estimation stage, enabling a cross-level fusion between scene understanding and quantitative measurement.

(27) $$v_{\mathrm{final}}=\hat{v}(y^{\lambda })-\beta A_{b}$$

where *β* is an empirical coefficient that converts the detected bubble area into an equivalent correction in the volume unit. The coefficient β was calibrated using a small set of samples with known reference readings collected under the fixed imaging setup. Specifically, *β* was estimated by minimizing the residual error between $v_{\mathrm{final}}$ and the ground-truth volume. Once the imaging setup was fixed, including the camera position, tube geometry, and illumination condition, *β* was kept unchanged for all subsequent tests. This is because the relationship between the detected bubble area and the induced volume offset is mainly governed by the fixed optical geometry and imaging scale. The adopted linear correction effectively captures the dominant bubble-related bias observed in practical measurements while retaining real-time efficiency and avoiding additional model complexity.

## 3. Experimental Protocols

**Figure 4:**
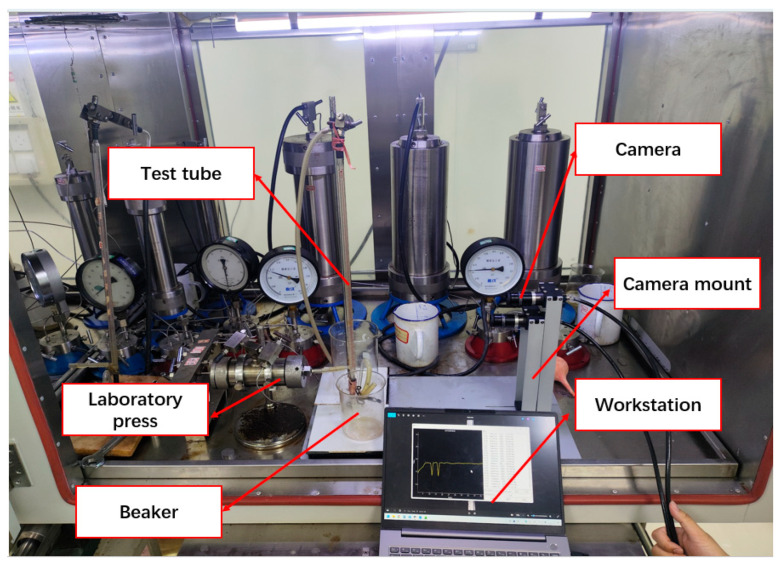
Experimental platform.

The experimental platform for oil–water interface measurement primarily consists of the following components: an industrial RGB camera (MER2-503-23GC, Daheng Imaging, Beijing, China) with a resolution of 2448 × 2048 pixels and a frame rate of 30 fps; a low-distortion C-mount lens (HN-0816-5M-C2/3, Daheng Imaging, Beijing, China) optimized for small field-of-view tube imaging; a custom-made rigid aluminum fixture that fixes the relative pose between the camera and the small-caliber test tube; and a high-performance workstation equipped with an Intel Core i9 CPU (Intel Corporation, Santa Clara, CA, USA) and an NVIDIA GeForce RTX 4060 GPU (NVIDIA Corporation, Santa Clara, CA, USA) for model training and real-time inference, as illustrated in Figure 4.

An industrial camera–lens assembly was used to acquire images of small-caliber tubes containing oil–water mixtures under practical laboratory conditions. The camera–tube geometry was kept fixed by a rigid fixture. The images include the true oil–water interface as well as typical artefacts, mainly tube-wall stains and small oil bubbles near the interface. These artefacts are explicitly detected by the proposed coarse stage to support robust interface localization and to enable bubble-related bias correction.

A dedicated dataset was collected across multiple tubes and three lighting conditions. According to the Tube ROI count in Table 2, the dataset contains 544 annotated images. This dataset supports the evaluation of the proposed method within the calibrated experimental setup, while broader generalization to different vessels, liquid systems, or illumination environments requires further validation. Each image was manually annotated with four classes: Tube ROI, liquid-surface cues, oil bubbles, and stain artefacts. The dataset was divided into training and validation subsets at a ratio of 8:2. YOLOv8n was trained on the training split and validated on the remaining subset, and the final detector was then fixed for all subsequent experiments, see Table 2.

To further verify the proposed method under practical conditions, sampled frames from the full shale-oil core experiment process were used for process-level evaluation. This experiment, conducted entirely independently of the training set, was used to test the model’s generalization ability. For each sampled image, YOLOv8n provides four-category detections, and the tube ROI is further analyzed by the fine stage, where chromatic and vertical-gradient cues are fused for interface localization under stain masking and cue consistency constraints. The validated interface row is then converted into a volume readout through graduation-based interpolation, followed by bubble-area-based correction. The corresponding ground-truth readings were obtained by manual readout of the tube graduations. In this way, the complete pressing process can be assessed through a sparse sequence of representative measurements, allowing validation of both robustness and reading stability in a realistic experimental environment.

## 4. Results and Discussion

**Figure 5:**
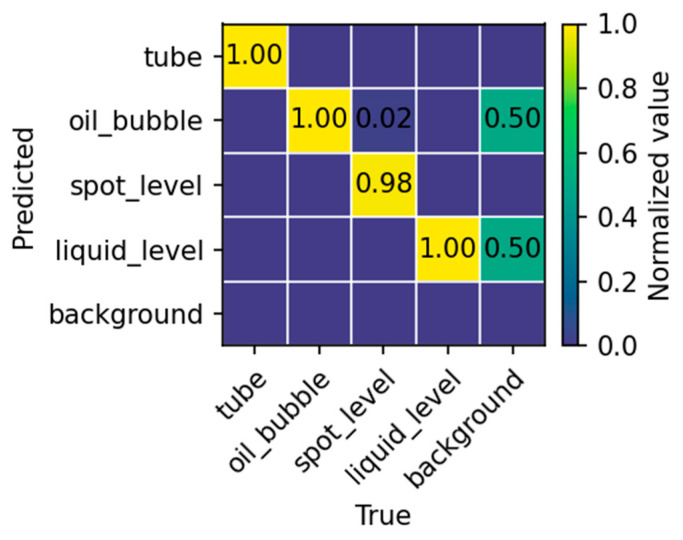
Coarse-stage detection results produced by the YOLOv8n model.

The proposed coarse-to-fine framework delivered stable oil–water interface localization across heterogeneous laboratory scenes, including uneven illumination, specular highlights, and tube-wall contamination. On the validation set, the coarse-stage detector achieved a precision of 0.96, a recall of 0.97, and an mAP@0.5 of 0.98, suggesting good detection performance under the current acquisition conditions. The confusion matrix in Figure 5 indicates that the four foreground classes remained well separated, especially for the tube ROI and liquid-surface cue, while only limited confusion was observed between stain artefacts and oil bubbles. Such an error distribution is favorable for the downstream task, because the semantic components most critical to subsequent interface localization are preserved in most cases. The remaining errors were mainly related to background over-response and to a small number of visually ambiguous cases. In particular, the images that were not correctly recognized were mainly those in which unbroken oil bubbles floated directly on the liquid surface, making the apparent boundary difficult to distinguish from the true oil–water interface. This ambiguity is also commonly encountered in practical experiments and represents one of the principal causes of the residual misclassification.

**Figure 6:**
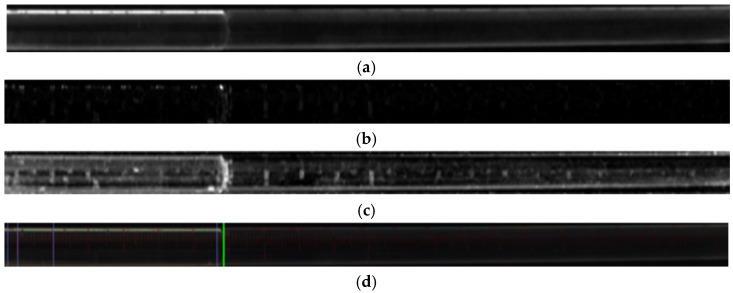
Visualization of the fine-stage oil–water interface detection process. (**a**) Chromatic-weighted brightness feature. (**b**) Vertical Sobel feature. (**c**) Fused feature combining chromatic and gradient cues. (**d**) Final detected oil–water interface (green line) overlaid on the tube ROI.

After ROI cropping, the fine-stage procedure yields an interface estimate that is visually coherent and consistent with the expected horizontal boundary geometry. Figure 6 presents the intermediate features and the final interface overlay. The chromatic-weighted brightness feature enhances photometric contrast near the phase boundary, whereas the vertical Sobel feature captures structural discontinuities in the vertical direction. Both cues are standardized at the ROI level and fused pixel-wise into a single response score feature, see Figure 6.

Table 3 separates the contributions of stain-aware processing and bubble compensation in a stage-wise manner. The original readout shows the largest deviation from reference, indicating that raw measurements are strongly affected by stain artefacts and bubble interference. After stain treatment, the error is markedly reduced, and bubble compensation further improves the result. Among the three stages, the bubble-compensated readout achieves the best performance, with the lowest MAE, RMSE, and maximum error, demonstrating the effectiveness of the proposed stage-wise correction strategy.

**Figure 7:**
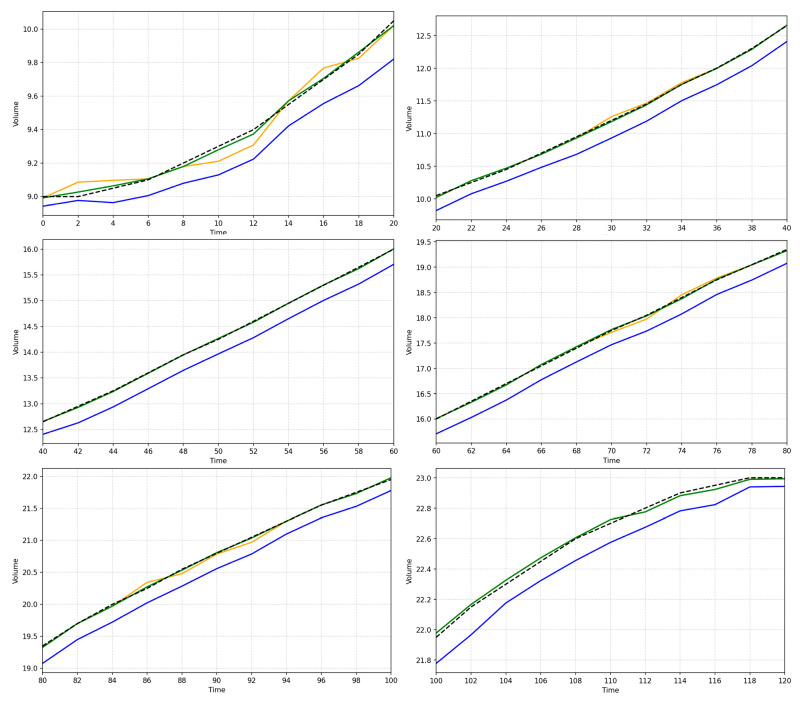
Comparison of original and corrected reading sequences in six segments of the shale oil pressing experiment. The blue, orange, and green solid lines denote the original, stain-treated, and bubble-compensated readings, respectively, while the black dashed line indicates the actual reading.

Figure 7 demonstrates the effectiveness of the correction stage over the entire shale oil pressing process. The plotted curves correspond to a continuous one-hour monitoring experiment, in which one frame was acquired every 30 s. Alongside the original readings, multiple compensated sequences are shown to illustrate the progressive integration of stain treatment and bubble compensation. Relative to the uncorrected estimates, the corrected results remain consistently closer to the actual readings throughout the process. Notably, during the middle phase of the experiment, when bubble accumulation gradually intensifies and causes stronger interference, the proposed method still preserves reliable correction performance. Moreover, stain compensation improves the smoothness of the readout curve by mitigating reflection-induced artefacts and suppressing non-physical local fluctuations.

The ablation results confirm that both Chromatic-Weighted Brightness Construction and Vertical-Gradient Feature Construction contribute to accurate and stable interface readout. Using Chromatic-Weighted Brightness only yields relatively large volume deviations, indicating that brightness-based chromatic discrimination alone remains sensitive to illumination variation and local appearance disturbances. In contrast, Vertical-Gradient Feature Construction only achieves smaller volume deviations, suggesting that vertical structural transitions provide a more reliable cue for interface localization. The best performance is obtained by combining Chromatic-Weighted Brightness with Vertical-Gradient Feature Construction, which demonstrates that the two types of cues are complementary in the fine-stage localization process.

Taken together, these results support a simple point: semantic coarse detection and signal-level fine localization are not redundant; they are complementary. The coarse stage stabilizes the measurement context, while the fine stage extracts a precise interface estimate from fused photometric and structural evidence. Coupled with graduation-based pixel-to-scale feature and bubble compensation, the system provides reliable volume readout in a compact laboratory setup and offers a practical basis for broader multiphase liquid monitoring, see Table 4.

Table 5 compares the coarse-stage ROI detection performance of the proposed YOLOv8n-based method with two conventional image-processing methods. The comparison was conducted using the same test dataset and the same evaluation protocol to ensure fairness. As shown in Table 5, the proposed YOLOv8n method achieves the highest Mean IoU of 0.97, outperforming Canny edge detection and Hough Transform, which obtain Mean IoU values of 0.82 and 0.88, respectively. This result indicates that YOLOv8n provides more accurate and stable coarse-stage localization of the measurement region.

In terms of computational efficiency, YOLOv8n also achieves the shortest runtime of 6.2 ms, compared with 7.5 ms for Canny edge detection and 17.8 ms for Hough Transform. Although Canny edge detection is computationally simple, its localization accuracy is more easily affected by weak edges, reflections, stains, and non-uniform illumination. Hough Transform improves the localization accuracy to some extent by introducing geometric constraints, but it requires higher computational cost and remains sensitive to incomplete or distorted boundaries. In contrast, YOLOv8n learns semantic features of the tube region and is therefore less dependent on manually defined edge or geometry assumptions. These results demonstrate that the proposed YOLOv8n-based coarse detection strategy achieves a better balance between detection accuracy and runtime efficiency, providing a more reliable basis for the subsequent fine-stage metrological refinement.

Table 6 compares the volume measurement accuracy and runtime efficiency of different methods. The runtime represents the complete single-frame processing time. Canny edge detection achieves 116.3 FPS, but it produces large measurement errors because it is sensitive to artefacts, stains, reflections, and weak edge responses. Hough Transform provides relatively better stability by introducing geometric constraints, but its parameter-space search leads to the lowest FPS of 52.9, and it can still be affected by environmental interference or distorted boundaries. Threshold segmentation achieves the highest FPS of 144.9 owing to its simple computation, but its accuracy is strongly affected by non-uniform illumination and local contrast variation.

**Figure 8:**
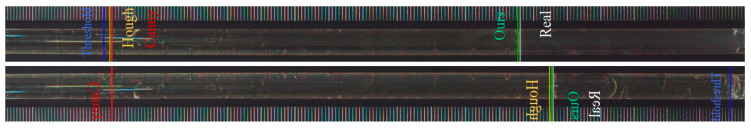
Visual comparison of volume readout results obtained by different methods.

Compared with these methods, the proposed method achieves the lowest MAE, RMSE, and maximum error, with values of 0.0159 mL, 0.0181 mL, and 0.0288 mL, respectively. Meanwhile, it maintains a processing speed of 69.9 FPS, which is sufficient for real-time automated oil–water interface readout. These results demonstrate that the proposed method provides a better balance between measurement accuracy, robustness, and runtime efficiency than conventional edge-, geometry-, and threshold-based methods. The qualitative results in Figure 8 further demonstrate that the proposed method provides more robust interface localization under artefacts, illumination variation, and environmental disturbances. The large errors of conventional methods mainly arise from failure cases in which the detected boundary deviates substantially from the true interface position.

For dark-colored oils with high opacity, the vertical-gradient feature may become less reliable in several cases. First, high opacity can reduce the visible contrast across the oil–water interface, causing the vertical intensity transition to become weak or blurred. Second, dark oils may absorb more light and generate local shadowing, which can produce misleading gradient responses unrelated to the true interface. Third, when the oil phase is visually close to the background or when the interface is affected by reflection and cylindrical-glass refraction, the SobelY response may shift from the true boundary to nearby artefact edges. In such cases, the vertical-gradient cue alone may fail to identify the correct interface.

**Figure 9:**
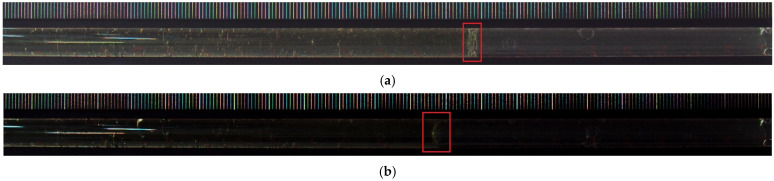
Typical challenging scenarios and failure cases. (**a**) Overlapping bubbles near the oil–water interface. (**b**) Ambiguous interface caused by weak contrast or boundary disturbance. The red boxes in (**a**) and (**b**) indicate overlapping bubbles and ambiguous interfaces, respectively.

Figure 9 presents two typical challenging scenarios. In the overlapping-bubble case shown in Figure 9a, bubble boundaries near the true oil–water interface may generate additional edge and chromatic responses, making it difficult to distinguish the true interface from bubble-induced disturbances. Although the proposed compensation strategy reduces the influence of isolated bubbles, severe overlap can still bias the estimated interface position. In the interface-ambiguity case shown in Figure 9b, weak contrast, reflection, or local surface disturbance makes the oil–water boundary visually unclear. This reduces the discriminative ability of chromatic and gradient cues and increases localization uncertainty. These results show that the proposed method remains limited under severe bubble overlap and ambiguous interface conditions, which will be addressed in future work.

From an application perspective, the proposed framework provides a feasible solution for automated oil–water interface readout in a fixed small-tube imaging setup. However, several limitations remain. The current validation is limited to a single oil–water system under a fixed imaging geometry. For liquids with different colours or optical contrasts, the chromatic-discriminative weights may need to be recalibrated, and the current colour-based cue may become less effective when the two phases have similar appearance or when dark-coloured oils are used. In addition, cylindrical-glass refraction may introduce an apparent vertical shift and local edge distortion of the oil–water interface, causing the chromatic or vertical-gradient response peak to deviate from the true physical boundary. Different glass materials may also change light transmission, surface reflection, and refractive distortion, thereby affecting fine-stage interface localization.

The coarse-to-fine framework is structurally transferable to tubes with different diameters and wall thicknesses, but its calibrated parameters are setup-dependent. Extending the method to new tube geometries would require recalibration or fine-tuning of the YOLO detector, graduation anchors, chromatic weights, image-to-volume mapping, and bubble compensation coefficient β. For more complex multiphase systems, such as oil–water–gas or emulsion-containing systems, bubbles, droplets, and irregular interfaces may interfere with the true liquid boundary, and additional phase-specific annotations, segmentation, or temporal-tracking modules may be required. Future work will focus on reducing calibration dependence and improving robustness across more diverse liquid systems, tube geometries, glass materials, and disturbance patterns.

## 5. Conclusions

This study presented a coarse-to-fine framework for readout-oriented oil–water interface measurement in small-caliber transparent test tubes. In the coarse stage, a lightweight YOLOv8n detector was used to establish the measurement context by identifying the tube ROI together with liquid-surface cues, stain artefacts, and oil bubbles. This semantic perception stage constrains the subsequent analysis to the physically meaningful measurement region and provides disturbance-related priors. In the fine stage, interface localization was performed within the cropped ROI by combining a Fisher-discriminative chromatic-weighted brightness cue with a SobelY-based vertical-gradient cue. Stain-aware row aggregation with effective-pixel compensation was further introduced to suppress unreliable responses, and the validated interface row was subsequently converted into a volume readout through graduation-based interpolation with additional bubble-related correction.

The experimental results indicate that the proposed design can provide a practically stable measurement pipeline under the tested laboratory conditions. Process-level evaluation on sampled frames from a complete shale-oil core pressing procedure showed that the framework can maintain consistent interface readout in the presence of illumination variation, stains, and bubble interference, with an MAE of 0.0159 mL and a maximum error below 0.03 mL. In addition, the stage-wise analysis and ablation results suggest that semantic coarse-stage constraints and fine-stage cue fusion are complementary: the former stabilizes the measurement context by restricting the search space and identifying disturbance cues, whereas the latter preserves metrological precision by performing local interface refinement within the constrained ROI. This separation prevents coarse semantic detection from being overburdened with pixel-level measurement and prevents fine-stage feature extraction from being disturbed by irrelevant background or artefact regions.

From an application perspective, the proposed framework is suitable for automated shale-oil analysis systems when the imaging setup is fixed, the tube geometry is known, the liquid pair is predefined, and bubble/stain disturbances remain within a manageable range. For different liquid systems, tube diameters, wall thicknesses, glass materials, or more complex multiphase conditions, the framework may still be structurally transferable, but recalibration or fine-tuning would be required. In particular, the YOLO detector, graduation anchors, chromatic weights, image-to-volume mapping, and bubble compensation coefficient should be re-estimated when the imaging geometry or optical properties change. Future work will focus on reducing calibration dependence and improving robustness across more diverse vessels, liquid systems, and disturbance patterns.

## Figures and Tables

**Table 1:** Detection categories used in the YOLOv8n coarse stage.

Category	Functional Role
Tube ROI	Overall tube boundary used to crop the measurement region.
Stain artefacts	Marks on tube walls used to suppress false boundary responses.
Oil bubbles	Floating oil droplets used for volume correction.
Liquid-surface cues	Near the oil–water boundary serving as structural hints.

**Table 2:** Dataset composition across scene types, and annotation categories.

Scene Type	Tube ROI	Liquid-Surface Cues	Oil Bubbles	Stain Artefacts
Bright illumination	181	181	501	181
Low-light	181	181	456	180
Mixed conditions	182	182	414	169

**Table 3:** Overall readout accuracy of different processing stages.

Stage	MAE (mL)	RMSE (mL)	Max Error (mL)
Original	0.2184	0.2333	0.3272
Stain-Treated	0.0273	0.0367	0.0943
Bubble-Compensated	0.0159	0.0181	0.0288

**Table 4:** Ablation study of the fine-stage feature construction methods.

Method	MAE (mL)	RMSE (mL)	Max Error (mL)
Chromatic-Weighted Brightness only	0.0316	0.0408	0.0869
Gradient Feature Construction only	0.0238	0.0305	0.0574
Fusion of Brightness and Gradient	0.0159	0.0181	0.0288

**Table 5:** Comparison of ROI Detection Methods in Accuracy and Runtime.

Method	Mean IoU	Runtime
Canny edge detection	0.82	7.5 ms
Hough Transform	0.88	17.8 ms
YOLOv8n (proposed)	0.97	6.2 ms

**Table 6:** Comparison of Volume Measurement Obtained by Different Methods.

Method	MAE (mL)	RMSE (mL)	Max Error (mL)	Runtime per Frame (ms)	FPS
Canny	9.2424	10.5627	18.6049	8.6	116.3
Hough	4.5223	7.5442	18.7132	18.9	52.9
Threshold segmentation	8.1404	9.0208	18.7531	6.9	144.9
Ours	0.0159	0.0181	0.0288	14.3	69.9

## Data Availability

The data presented in this study are available from the corresponding author upon reasonable request, subject to the data security policy of Daqing Oilfield.

## Abbreviations

The following abbreviations are used in this manuscript:

ROI	Region of Interest
YOLO	You Only Look Once
RGB	Red, Green, Blue
MAE	Mean Absolute Error
RMSE	Root Mean Square Error
